# Creutzfeldt-Jakob disease: recent developments

**DOI:** 10.12688/f1000research.12681.1

**Published:** 2017-11-27

**Authors:** Graeme Mackenzie, Robert Will

**Affiliations:** 1 National CJD Research & Surveillance Unit, Bryan Matthews Building, Western General Hospital, Edinburgh, EH4 2XU, UK

**Keywords:** Creutzfeldt-Jakob, prion disorder, variant CJD, diagnosis

## Abstract

Creutzfeldt-Jakob disease (CJD) is a rare prion disorder that has been the subject of both professional and public interest following the identification of variant CJD as a zoonotic disorder. There have been recent advances in diagnostic techniques, including real-time quaking-induced conversion and magnetic resonance imaging brain scan, that have allowed more accurate case recognition in all forms of CJD. Although the epidemic of variant CJD is clearly in decline, prevalence studies suggest that it may be premature to be complacent about concerns for public health.

## Introduction

Creutzfeldt-Jakob disease (CJD) belongs to a family of fatal degenerative disorders of the nervous system known as transmissible spongiform encephalopathies or prion diseases, which affect both animals and humans ^1^. The term prion, derived from *proteinaceous infectious* particle, was coined by Stanley Prusiner after the identification of the disease-associated protein ^2^. The normal prion protein, PrP ^C^, is present in all mammalian species and is encoded by the prion gene ( *PRNP*) on human chromosome 20. The function of prion protein has not been established. Prion diseases are characterised by the deposition of PrP ^Sc^, an abnormally misfolded isoform of the native prion protein, within the nervous system. The mechanism for triggering this conformational change is not known, but the accumulation of this abnormal prion protein leads to neuronal degeneration, astrocytic gliosis, and spongiform change, resulting in a uniformly fatal neurological disorder ^3^.

The human prion disorders are heterogeneous with different phenotypes, epidemiology, and pathogenesis. Sporadic CJD (sCJD) is the commonest human prion disease, accounting for around 85% of cases; 10–15% are associated with mutations of *PRNP* and 1% are iatrogenic, most frequently associated with prior treatment with human pituitary-derived hormones or human dura mater grafts. Variant CJD (vCJD) is a novel human prion disease which occurs predominantly in the UK and has been linked to the consumption of beef products contaminated with the agent of the cattle disease, bovine spongiform encephalopathy (BSE) ^4– 6^.

sCJD has a very rapid disease course; mean survival is six months. Indeed, over 90% of patients die within a year of symptom onset. The peak incidence is in the seventh decade, and younger (20–40s) or older (>80) cases are less common ^7^. The favoured hypothesis is that sCJD is a spontaneous neurodegenerative disease, resulting from either a somatic *PRNP* gene mutation or a random structural change in the PrP protein causing the formation of PrP ^Sc^ ^2^. An environmental source for the disease is not supported by epidemiological studies.

There have been recent developments in diagnostic investigations in CJD, and, although the vCJD outbreak is in decline, there are continuing concerns for public health in relation to the prevalence of infection in the normal population. These issues and the potential relevance of prion diseases to other neurodegenerative disorders are the main topics of this article.

## Diagnosis

There is considerable variability in the way in which CJD can present clinically, which can make the initial diagnosis difficult. This heterogeneity of clinical presentation is linked, at least in part, to variations in the genotype at codon 129 of the prion protein gene and the type of PrP ^Sc^ deposited in the brain. The genotype at codon 129 can be methionine homozygous (MM), valine homozygous (VV), or heterozygous (MV), and in the UK population, the normal codon 129 distribution has been reported as 39% MM, 50% MV, and 11% VV ^8^. Two biochemically distinct forms of PrP ^Sc^, type 1 and type 2, can be deposited in the brain.

The classic clinical features of sCJD are rapid cognitive decline, ataxia, and myoclonus terminating in an akinetic mute state. The diagnosis of CJD is dependent upon assessment of clinical features together with specialist investigations. There has been an evolution in the diagnosis of CJD in recent years with the identification of new diagnostic tests, and this has been reflected in changes to the formal diagnostic criteria for sCJD used in the European Union (EU).

### Magnetic resonance imaging

**Figure 1.**
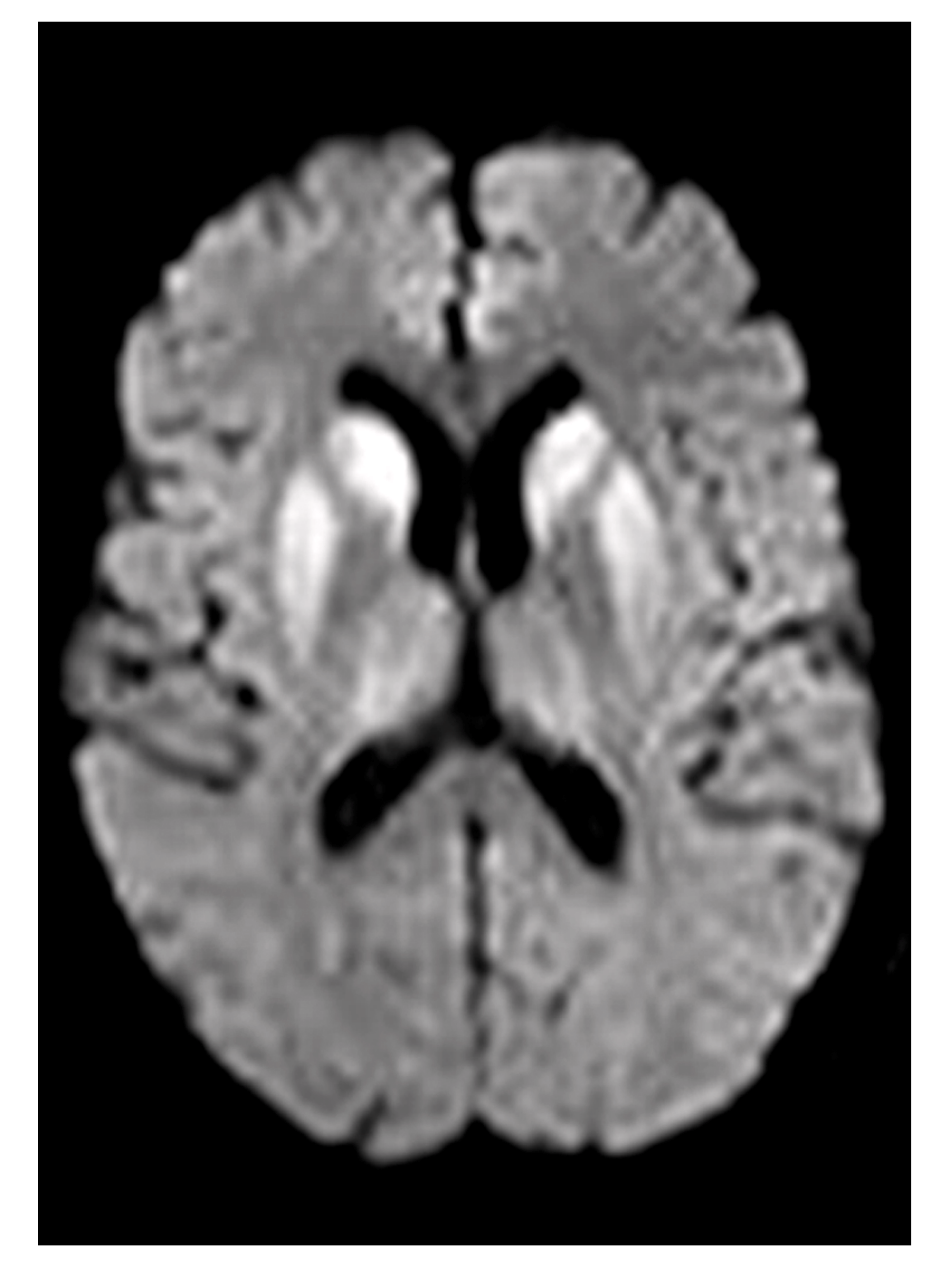
Magnetic resonance imaging of sporadic Creutzfeldt-Jakob disease. Diffusion-weighted image at the level of the basal ganglia demonstrates marked symmetrical hyperintensity in the caudate head and putamen with less marked affection of the thalami. Image courtesy of David Summers, Western General Hospital, Edinburgh, UK.

***Magnetic resonance imaging in sporadic Creutzfeldt-Jakob disease.*** Magnetic resonance imaging (MRI) is the most useful investigation in sCJD, as it is highly sensitive and specific as well as widely available and relatively non-invasive ^9^. The classic MRI findings in sCJD, including high signal in the caudate, putamen, or cortex (or a combination of these) on diffusion-weighted imaging (DWI) and fluid-attenuated inversion recovery (FLAIR) sequences, are present in about 80% of cases ^9^. DWI is more sensitive at detecting early cortical and subcortical changes ( Figure 1).

**Figure 2.**
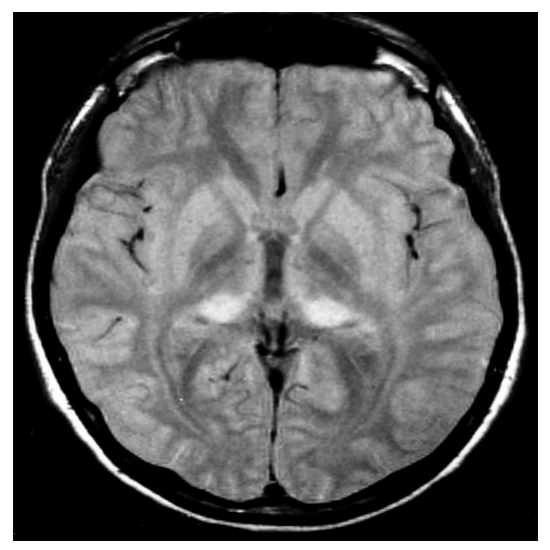
Pulvinar sign of variant Creutzfeldt-Jakob disease. Axial fluid-attenuated inversion recovery image demonstrates symmetrical hyperintensity of the posterior thalamic nuclei. Image courtesy of David Summers, Western General Hospital, Edinburgh, UK.

***Magnetic resonance imaging in variant Creutzfeldt-Jakob disease.*** Symmetrical hyperintensity in the posterior thalamus, relative to the anterior putamen, on T2-weighted or FLAIR MRI is characteristic of vCJD and is known as the pulvinar sign ( Figure 2) ^10^. This finding is very rare in other types of prion disease ^11^ and is reported to have a sensitivity of 78–90% and a specificity of 100% for vCJD in the right clinical setting ^5^. The mediodorsal thalamic nucleus may also be involved and in combination with pulvinar hyperintensity produces an appearance coined the ‘hockey stick sign’. In 86 neuropathologically confirmed cases, involvement of the caudate nucleus on FLAIR MRI was shown in 40%, the putamen in 23.3%, and the periaqueductal grey matter in 83.3% ( Figure 2) ^10^.

Until recently, all definite cases of vCJD have been MM at codon 129, which may account for the observed similarity of MRI findings in these cases when compared with other human prion diseases. This concept has been challenged, however, with the recent description of a neuropathologically confirmed case of vCJD with a heterozygous genotype at codon 129 ^12^. The MRI findings in this case demonstrated restricted diffusion in the basal ganglia, hypothalamus, insular cortexes, and medial thalami but not in the pulvinar nuclei.

### Real-time quaking-induced conversion

A number of cerebrospinal fluid (CSF) biomarkers, including 14-3-3, S100b, and tau, have been found to be elevated in patients with CJD. These proteins have limited specificity but can be diagnostically useful in the appropriate clinical context ^13^.

**Figure 3.**
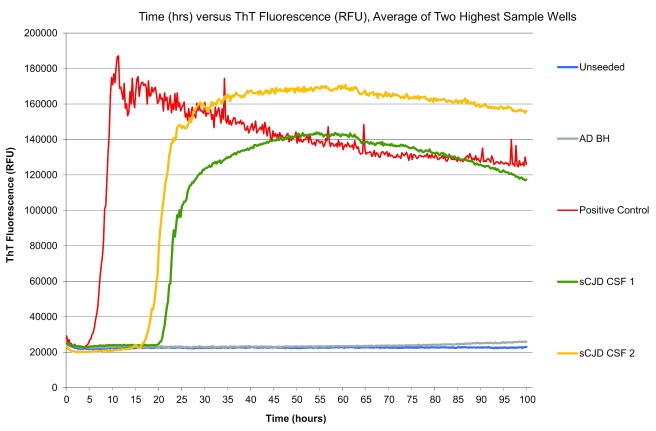
Real-time quaking-induced conversion (RT-QuIC) responses from reactions seeded with cerebrospinal fluid (CSF) from two sporadic Creutzfeldt-Jakob disease (sCJD) cases and a positive sCJD CSF control. The RT-QuIC from an unseeded reaction and a reaction seeded with brain homogenate from Alzheimer’s disease are also shown. Image courtesy of Neil McKenzie, University of Edinburgh, Edinburgh, UK. AD BH, Alzheimer’s disease brain homogenate; RFU, relative fluorescence units; ThT, thioflavin T.

Real-time quaking-induced conversion (RT-QuIC) is a relatively new CSF test which has shown remarkably high sensitivity and specificity in recent studies (sensitivity of 85.7% and specificity of 100%) in sCJD ^14^. The technique involves using recombinant PrP as a substrate which is then induced to aggregate by adding CSF containing PrP ^Sc^. Thioflavin T then binds to the aggregated PrP ^Sc^, causing a change in the thioflavin T emission spectrum and enabling the reaction to be monitored in real time ( Figure 3). It is also a useful investigation for some genetic forms of CJD ^15^.

***Real-time quaking-induced conversion olfactory mucosa and cerebrospinal fluid.*** The use of olfactory mucosa (OM) nasal brushings combined with RT-QuIC has been shown to improve the diagnosis of sCJD ^16^. In a recent study ^17^, the combined utilisation of CSF and OM RT-QuIC produced an overall specificity and sensitivity of 100% for the diagnosis of sCJD. OM RT-QuIC is limited, however, by the technical expertise required to obtain appropriate specimens. Although RT-QuIC is clearly a useful test for sCJD, the assay in its current form does not amplify the PrP ^Sc^ in vCJD ^18^.

### Blood and urine tests in variant Creutzfeldt-Jakob disease

In an attempt to develop a better pre-mortem test for vCJD, a number of studies have attempted to detect the abnormal PrP ^Sc^ in vCJD in blood and urine by using protein misfolding cyclic amplification (PMCA). In 2014, Moda *et al*. successfully detected PrP ^Sc^ in the urine of patients with vCJD ^19^. The study looked at 238 urine samples from patients with vCJD, sCJD, or genetic prion diseases and healthy controls in a blinded study. Using PMCA, they were able to amplify the PrP ^Sc^ to detectable amounts with a sensitivity of 93% and a specificity of 100% in vCJD. All the sCJD and control samples were negative.

Similar studies using PMCA have led to the detection of PrP ^Sc^ in plasma from clinical vCJD as well as in pre-clinical blood samples taken from two vCJD patients who had donated blood prior to the onset of symptoms ^20, 21^. This is the first time that a test has successfully identified PrP ^Sc^ in pre-clinical samples, and it may have the potential to be applied as a screening tool in transfusion medicine, although further validation is necessary.

### Electroencephalogram

The electroencephalogram (EEG) is now a less useful investigation given the advent of MRI and CSF tests. Nonetheless, it remains an important, non-invasive surrogate marker for sCJD. The typical EEG appearances in sCJD are periodic, triphasic sharp wave complexes. These changes in the right clinical setting have a 90% specificity for CJD ^9^ but are known to occur in other conditions, such as end-stage Alzheimer’s disease, Lewy body dementia, and metabolic encephalopathies ^22, 23^.

### Diagnostic criteria for sCJD

The current diagnostic criteria used in the EU have been updated to include the cortical changes on MRI and RT-QuIC and are shown in Table 1.

**Table 1.** Diagnostic criteria for surveillance of sporadic Creutzfeldt-Jakob disease from 1 January 2017.

1.1 DEFINITE: Progressive neurological syndrome AND Neuropathologically or immunohistochemically or biochemically confirmed
1.2 PROBABLE: 1.2.1 I + two of II and typical electroencephalogram ^a^ OR 1.2.2 I + two of II and typical magnetic resonance imaging brain scan ^b^ OR 1.2.3 I + two of II and positive cerebrospinal fluid (CSF) 14-3-3 OR 1.2.4 Progressive neurological syndrome and positive real-time quaking-induced conversion in CSF or other tissues
1.3 POSSIBLE: I + two of II + duration <2 years
I Rapidly progressive cognitive impairment
II A Myoclonus B Visual or cerebellar problems C Pyramidal or extrapyramidal features D Akinetic mutism

^a^Generalised periodic complexes. ^b^High signal in caudate/putamen on magnetic resonance imaging brain scan or at least two cortical regions (temporal, parietal, occipital) on either diffusion-weighted imaging or fluid-attenuated inversion recovery.

## Epidemiology

The incidence of sCJD is frequently quoted as one per million population per annum. However, there has been a consistent gradual increase in mortality rates in the UK and in many other countries in which systematic surveillance is undertaken. This may relate to changes in population demographics, with an increase in the numbers of individuals in the age cohorts with a high incidence of sCJD, and improved case ascertainment as a result of increased awareness and more sensitive diagnostic investigations. Mortality rates of 1.5–2 cases per million may be more realistic on current evidence.

**Figure 4.**
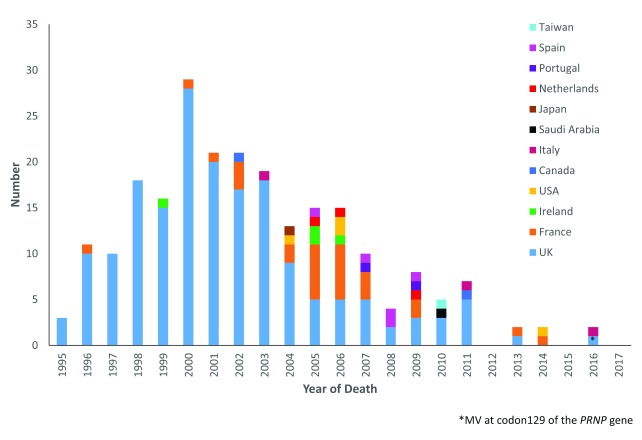
Variant Creutzfeldt-Jakob disease cases by year and country. MV, methionine valine heterozygous; *PRNP*, prion gene.

The vCJD epidemic is in marked decline both in the UK and internationally ( Figure 4). Until recently, all tested cases of definite and probable vCJD had been MM at codon 129 of *PRNP*, but in 2015 a confirmed case with an MV genotype was identified ^12^. This raises the possibility of a further outbreak, although it is likely that should this occur the number of cases will be relatively limited and probably lower than those of the primary epidemic in the MM population. The UK population were exposed to a significant level of BSE infectivity in the food chain in the 1980s and early 1990s, and the relatively small scale of the vCJD epidemic may imply a significant barrier to infection between bovines and humans ^24^.

Secondary transmission of vCJD through blood transfusion is now established with three clinical cases of vCJD linked to the transfusion of non-leucodepleted red blood cells derived from individuals who themselves went on to develop vCJD ^25^. In addition, a recipient of an implicated transfusion who died of a non-neurological disorder was found to have PrP ^Sc^ positivity in the spleen, suggesting pre-clinical infection ^26^. This individual was a codon 129 heterozygote, and laboratory transmission studies demonstrated prion infectivity in the spleen with characteristics similar to those of primary cases.

The prevalence of vCJD infection in the general UK population has been studied by examining routine appendicectomy samples by immunocytochemistry. Positive samples were identified in 19 out of 33,115 appendices, leading to an estimated prevalence of 1 in 4,000 in the general population ^27^. This is a matter of concern, as there is no clinical evidence of infection prior to neuro-invasion in prion diseases and the mean incubation period in vCJD has been estimated to be 15 years. A more recent study examined appendix samples from before 1980, prior to the presumed start of the BSE epidemic, and in those born after 1996, after which human exposure to BSE was thought to be minimal. Surprisingly, 7 out of 15,959 of these samples were positive, suggesting either a more extended period of human exposure to BSE or perhaps that positivity may not imply infection with vCJD ^28^.

## Other misfolding protein disorders

In addition to prion diseases, there are a number of diseases associated with protein misfolding. These conditions are associated with the accumulation of oligomers of A-beta, tau, and alpha-synuclein and include Alzheimer’s disease, Parkinson’s disease (PD), and Huntington’s ^29^. The identification of alpha-synuclein deposits in a foetal graft in patients with PD ^30, 31^ led to the hypothesis that there may be the potential of seeding of protein from abnormal to normal tissue and subsequently there have been extensive studies in laboratory animals that suggest that this may occur in many protein misfolding disorders ^32^. The presence of beta-amyloid deposits in the brains of human growth hormone recipients and not in age-matched controls has added to concerns about parallels between prion diseases and other neurodegenerative diseases ^33, 34^. Whether this is an important issue for public health is uncertain and there may be a difference between seeding of protein and the actual transmission of a disease. One epidemiological study, for example, has shown no evidence of transfusion transmission of a number of neurodegenerative disorders, including Alzheimer’s disease ^35^.

## Conclusions

The diagnosis of CJD has improved in recent years with the advent of improved brain imaging and the development of specific CSF tests in sCJD and the potential for diagnostic tests in plasma and urine in vCJD. The public health concerns raised by the BSE epidemic have clearly decreased as new cases of vCJD have become a rarity in both the UK and other countries. However, the potential prevalence of infection in the general population remains a matter of concern and the parallels between prion diseases and other protein misfolding disorders are an area of active and continued research.
